# Supplementary material for: Learning curve and surgical outcome of robotic assisted colorectal surgery with ERAS program
Source: Sci Rep. 2022 Nov 29;12:20566. doi: 10.1038/s41598-022-24665-w (PMC9709162; doi:10.1038/s41598-022-24665-w)
Supplement: Supplementary file 2 — Supplementary Information 2. [file 41598_2022_24665_MOESM2_ESM.zip › RAL Raw data-Ñ[▒K/049/OP_ñw╝╨░O▒Kñσ.pdf]

正式摘要(電子簽章：00000206626198)

W95 / W95

臺中榮民總醫院

Operation Note

[redacted]

- 手術日期：2020/12/31
- 手術醫師：VS 蔣鋒帆，CR 陳映菁，CR 江世偉
- 執行麻醉：VS 陳永銘
- Anesthesia : GE
- Position : Lithotomy
- Aseptic agents : Soap , Betadine Alcohol , Betadine Aqua

Diagnosis

- Pre-operative diagnosis : Malignant tumor of sigmoid colon
- Post-operative diagnosis : Ditto.

Surgical Indication

- curative treatment

Operation

- 1. Robotic (XI) assisted anterior resection with end-to-end anastomosis and small intestine segmental resection with side-to-side anastomosis.
  2. Intraoperative colonoscopy : (-).
  3. High ligation (+) : At IMA root (Via hemolok). IMV was also ligated via hemolok.
  4. Take down from the splenic flexure : (-)
  5. Robotic endoGIA (60-4.3)*1 + EEA 29 mm (Echelon circular powered stapler) to complete end-to-end anastomosis.
  6. Proximal cut end: 3cm, distal cut end: 9 cm
  7. 11cm jejunum was resected, mesentary of the segment of small intestine was ligated, mesetary defect repair(+)
  8. No drainage tube was inserted
  9. ADHESION BARRIER FILM was placed below the extended wound

Finding

- 1. A 1.5*1.5 cm tumor at S-colon, which invaded to the muscular layer, but didn't penetrate through the serosa. No enlarged lymph nodes at mesorectum, cT2N0M0.
  2. A 3.5*2.5 cm submucosal tumor at jejunum, no mucosal lesion was noted. 11cm jejunum was resected.
  3. No other gross tumor nor polyps was found
  4. There was no obvious visible liver metastasis, no visible peritoneal seeding
  5. No obvious ascites was noted
  6. Bilateral doughnuts : intact ; Air leak test: Done and no air leak was found.

Procedure

- 1. General endotracheal anesthesia was induced and then the patient was placed in modified Trendelenburg lithotomy position.
  2. Preoperative antibiotics were given. A Foley catheter and nasogastric tube were placed.
  3. The abdomen was prepped and draped in standard sterile fashion.
  4. A small vertical incision was made para-umbilicus and a 8 mm trocar was inserted into the peritoneal cavity by open method. Peumoperitoneum was established with CO2 inflation.
  5. Laparoscopy was inserted and then other two 8mm trocars were inserted over LUQ, RLQ of abdomen. Another 12mm trocars was inserted over RLQ area, and the other assist-port 5mm troca was inserted on RUQ. The operative findings were mentioned as above.

- 6. The sigmoid colon was held by grasper and the peritoneum overlying the mesentery was scored with electrocautery.
  7. The inferior mesentery artery was identified, ligated above the left colic artery with robotic clips, and transected. IMV was also ligated via robotic clip and then transected.
  8. Dissection was done above the Gerota's fascia and toward the peritoneal attachments.
  9. The colon was mobilized from retroperitoneum overlying the sacral promontory and into the pelvis.

  10. Distal descending colon and s-colon were freely mobilized after the mesoclon was all dissected.
  11. The incision was then extended laterally and medially above the peritoneal reflection.
  12. The RS colon was divided with robotic endoGIA (60-4.3) X1.
  13. Mesentary of small intestine tumor was grasped by locked forcep, so as rescted colon stump.
  14. The paraumbilical wound of abdomen was further extended. We approached into the peritoneal cavity by open method.
  15. Small intestine was pulled out and mesentary was ligated, GIA 80-3.8mm*2 was used for side-to-side anastomosis. Then the small intestine was pushed back into peritoneal cavity.
  16. The distal s-colon was then taken out for carefully inspection to make sure it has an excellent blood supply. The mesenteric fat was gently cleared from the terminal 2-3 mm of the bowel ends.
  17. The field was isolated with moist pad and the bowel opened and sized. It was determined that a 29 mm circular stapler (Echelon circular powered stapler) could be accommodated. Purstring clamp and 1-0 prolene were used to clamp end of residual D-colon, Kocher clamp was used to clamp specimen side, and then the colon was transected. The anvil was inserted into the lumen of the colon, and fixed with mersilk (For later end to end approach)
  18. The colon was then put back to the abdominal cavity and pneumoperitoneum was constructed again.
  19. The anal canal was irrigated with distil water and then the circular stapler was introduced through the anus.
  20. The proximal bowel with anvil in place (end) was engaged with the circular stapler (End) and the stapler was then fired. Two complete donuts were obtained and the distal one was submitted to pathology for margin confirmation.
  21. The anastomosis was tested for leaks with irrigation solution and air introduced through the rectum and was found to be patent and intact.
  22. The bleeder was checked thorughly again.
  23. The trochar fascia wounds were all then closed with interrupted sutures via 1-O Vicryl.
  24. ADHESION BARRIER FILM was placed below the central abdominal wound.
  25. The extended fascia wound was closed with interrupted sutures via Num.1 Vicryl.
  26. Massive N/S was applied to irrigate the wound.
  27. The wound was further closed with 3-O Vicryl, and the skin was applied with SurgiSeal.
  28. The patient tolerated the procedure well and was brought to the POR in stable condition.
- Estimated blood loss: minimum
- 器械數量: 正確
- 紗布數量: 正確

Image

- PAINT
  - colon & rectum

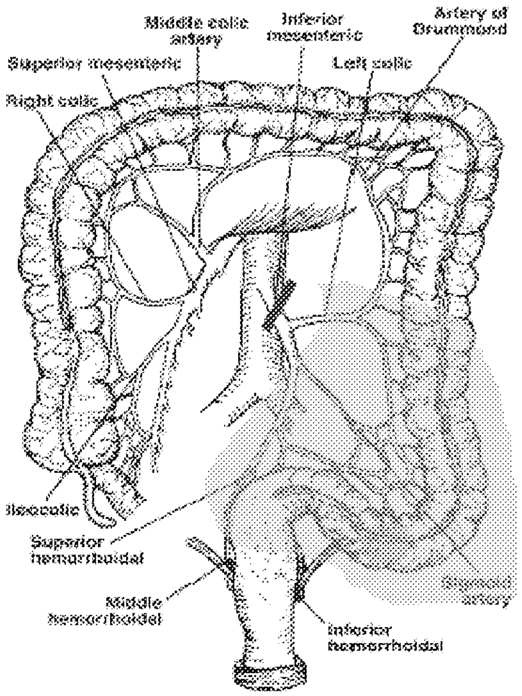

- abdomen

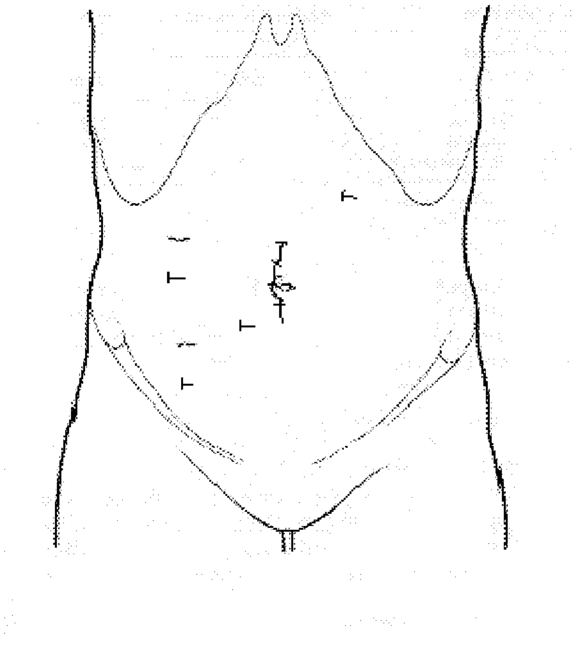

主任 2062F蔣鋒帆 / 醫師 2303K陳映菁

正式摘要(電子簽章：000000206626198)

W95 / W95

doctorRank:B
